# Unravelling the virome in birch: RNA-Seq reveals a complex of known and novel viruses

**DOI:** 10.1371/journal.pone.0221834

**Published:** 2020-06-26

**Authors:** Artemis Rumbou, Thierry Candresse, Armelle Marais, Laurence Svanella-Dumas, Maria Landgraf, Susanne von Bargen, Carmen Büttner

**Affiliations:** 1 Albrecht Daniel Thaer-Institute, Faculty of Life Sciences, Humboldt-Universität zu Berlin, Berlin, Germany; 2 UMR 1332, Biologie du Fruit et Pathologie, INRA, Univ. Bordeaux, CS, Villenave d’Ornon, Bordeaux, France; Oklahoma State University, UNITED STATES

## Abstract

To unravel the virome in birch trees of German and Finnish origin exhibiting symptoms of birch leaf-roll disease (BRLD), high-throughput sequencing (HTS) was employed. In total five viruses, among which three were so far unknown, were detected by RNAseq. One to five virus variants were identified in the transcriptome of individual trees. The novel viruses were genetically—fully or partially—characterized, belonging to the genera *Carlavirus*, *Idaeovirus* and *Capillovirus* and are tentatively named *birch carlavirus*, *birch idaeovirus*, and *birch capillovirus*, respectively. The recently discovered birch leafroll-associated virus was systematically detected by HTS in symptomatic seedlings but not in symptomless ones. The new carlavirus was detected only in one of the three symptomatic seedlings. The novel putative *Capillovirus* was detected in all seedlings—irrespective of their BLRD status—while the *Idaeovirus* was identified in a plant without leaf symptoms at the time of sampling. Further efforts are needed to complete Koch’s postulates and to clarify the possible association of the detected viruses with the BLR disease. Our study elucidates the viral population in single birch seedlings and provides a comprehensive overview for the diversities of the viral communities they harbor, to date.

## Introduction

The wide application of HTS technologies has significantly facilitated the discovery and characterization of viral agents in woody hosts, including trees, surpassing the limitations of more traditional approaches [1–3]. This has led to the identification of known and so far unknown viruses and provided novel insight into the virome of a range of several woody species. Regarding fruit trees, in the last five years, HTS use has led to the discovery of many new viruses [4–6]. The analysis of the RNA viromes of six peach trees identified, for example, up to six viruses and viroids in each tree [7]. The same situation has occurred in grapevine [8,9] and HTS data showed that grapevine plants infected by grapevine Pinot gris virus may acquire very complex viromes [10]. Building on these success stories, the application of HTS techniques for routine virus detection has gained momentum.

In birch (*Betula sp*.), the use of HTS approaches has led to the discovery of a novel virus, birch leafroll-associated virus (BLRaV, *Badnavirus*, *Caulimoviridae*), in trees from Germany and Finland affected by the birch leaf-roll disease (BLRD) [11]. This disease is observed throughout Europe [12–15] and could significantly reduce the tree’s photosynthetic capacity and contribute to decline [16]. Due to the correlation between BLRaV presence and BLRD-related symptoms, this virus is now considered a possible candidate in the etiology of BLRD.

In the present study, we aimed to provide additional knowledge on viruses in birches, as well as to investigate the composition of the virome of single trees. The exhaustive collection of nucleic acid sequences deriving from viral agents from five birch seedlings of German and Finnish origin are described. A complex of known and novel viruses and of diverse variants of those agents was found to infect the tested samples. Apart from the already described viruses, novel viral sequences from the genera *Carlavirus*, *Idaeovirus* and *Capillovirus* were identified and novel viruses were—fully or partially—genetically characterized. However, HTS is a qualitative method. Therefore, it is not adequate for attributing causal roles to some of the pathogens identified. To study the etiology of the disease and possible synergistic effects between the identified viral agents, further studies involving infectivity tests with infectious clones in greenhouse experiments will be necessary.

## Materials and methods

### RNA-Seq and sequence assembly

**Fig 1:**
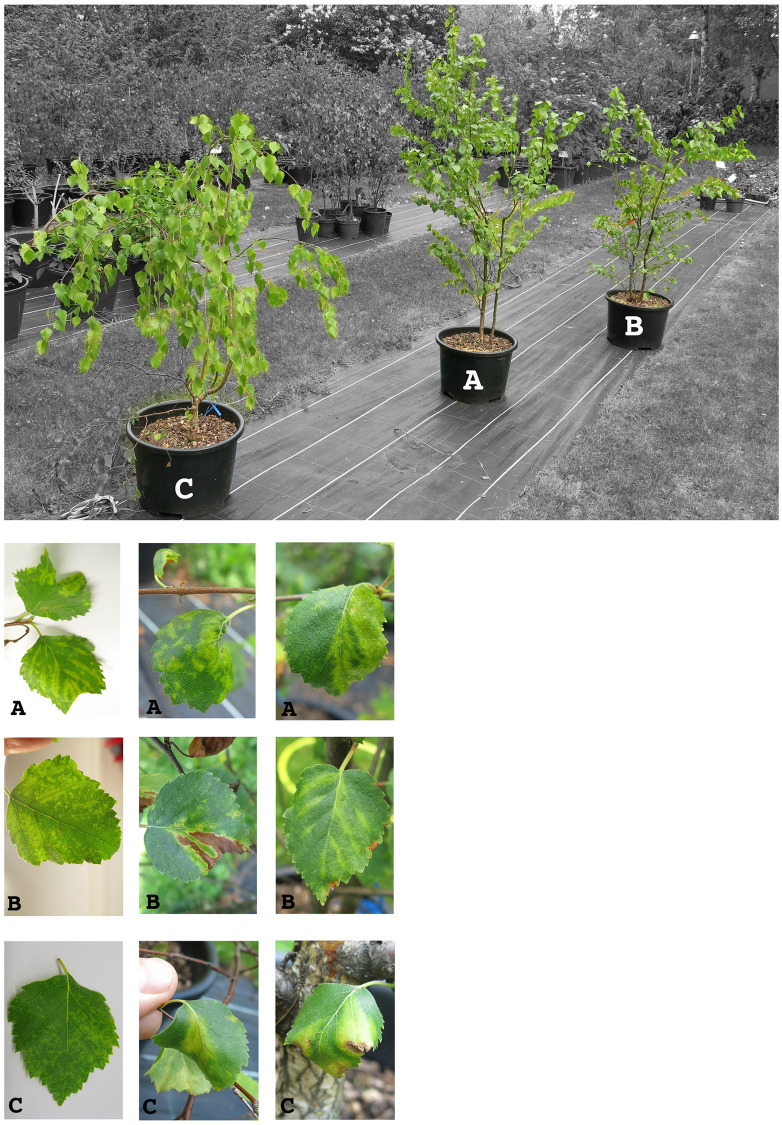
Leaf symptoms exhibited on the grafted birch seedlings BpubFinn407501_3A (A), BpubFinn407507_3I (B) and BpenGer407526_5M (C). Symptoms on seedling A: Leaf-roll, chlorotic vein banding, mottling and vein banding. Symptoms on seedling B: Mottling, leaf necrosis (final stage developed from chlorosis), vein chlorosis. Symptoms on seedling C: Leaf-roll, mottling, leaf roll and vein banding, and vein banding with necrosis.

Two twigs originating from an urban *Betula pubescens* donor tree (Bpub3) with severe BLRD leaf symptoms (vein banding, leaf chlorosis and necrosis, leaf rolling) from Rovaniemi (Finland) were grafted on two non-symptomatic *B*. *pubescens* rootstocks, generating grafted seedlings BpubFinn407501_3A and BpubFinn407507_3I. One twig originating from a *B*. *pendula* tree (Bpen 5) from Berlin, Germany, which was also exhibiting BLRD symptoms, was grafted on a non-symptomatic *B*. *pendula* rootstock, generating grafted seedling BpenGer407526_5M. One twig originating from the *B*. *pendula* tree MO197 from Berlin, Germany, not exhibiting symptoms at the time of the grafting, was grafted on a non-symptomatic *B*. *pendula* rootstock, generating the grafted seedling BpenGerM0197542. One CLRV-negative and symptomless birch seedling, the *B*. *pubescens* (BpubGer4), obtained from the same German nursery (nursery Reinke GbR Baumschulen, Rellingen, Germany), was used as a negative control. The following growing season, symptoms similar to the ones exhibited by the donor trees could be observed on three grafted birches already at the beginning of May and developed further until the end of September (Fig 1). No symptoms were observed on the symptomless *B*. *pubescens* seedling, while ambiguous symptoms were exhibited on the seedling BpenGerM0197542.

In 2014, pooled samples of five symptomatic leaves (Fig 1) randomly selected from the seedlings canopy were used for total RNA isolation. Similar leaf pools obtained from the symptomless seedlings were used in parallel. Total RNA isolation, cDNA synthesis and preparation for RNA-Seq analysis with the Illumina HiSeq2500 system are fully described in Rumbou et al., 2018 [11]. Paired-end 100 bp-long sequence reads corresponding to a 50–100 Mb data/sample were generated. All HTS data processing and analysis were performed using CLC Genomics Workbench version 7.0.4. Reads were first submitted to quality filtering and trimming. The resulting cleaned reads were then assembled into contigs that were finally annotated by BLASTN and BLASTP against the GenBank database.

### Taxonomic analysis of the metagenome

**Fig 2:**
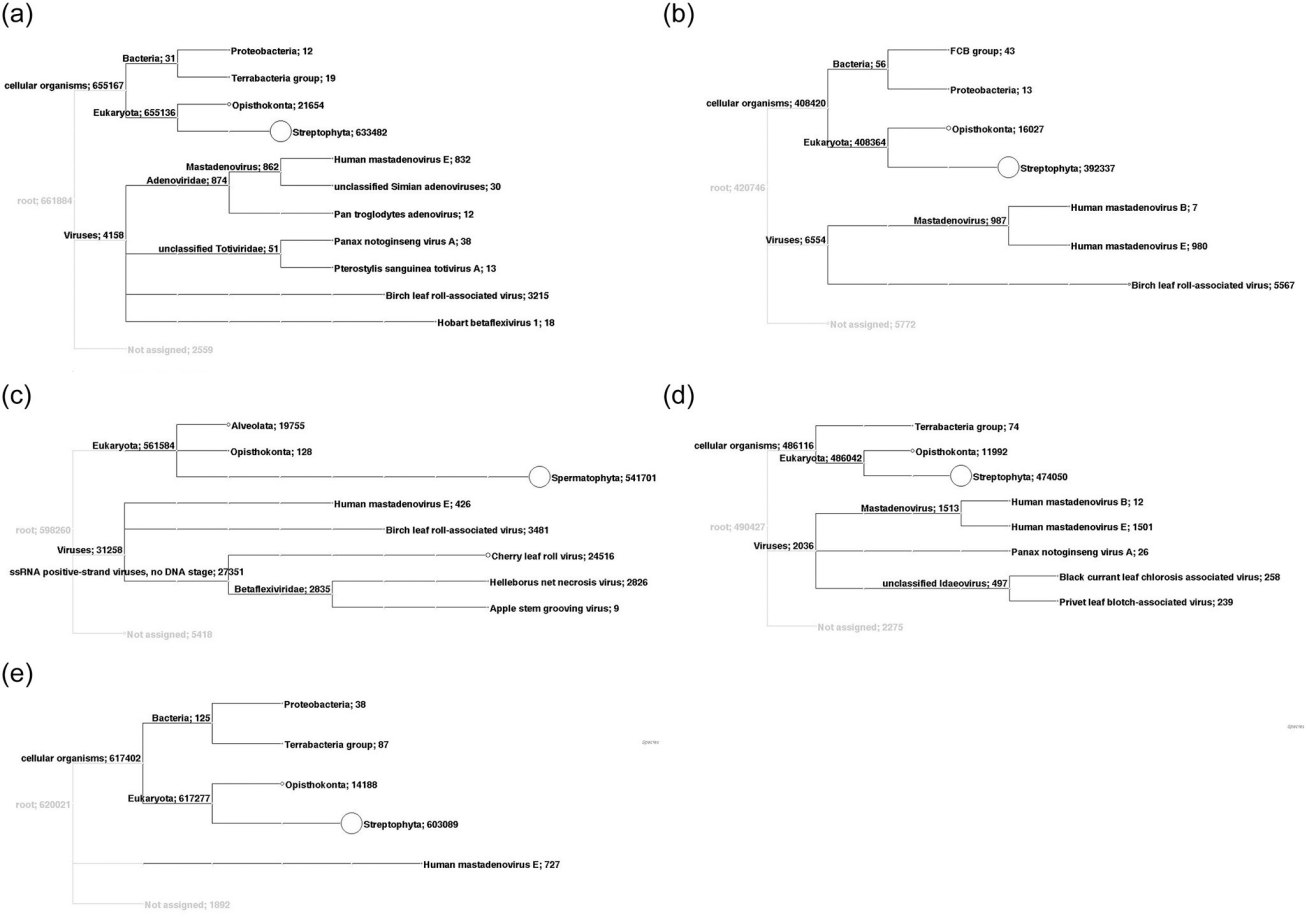
Taxonomical content of the birch samples analyzed by RNA-Seq with focus on the virome. A. symptomatic birch BpubFinn407501_3A, B. symptomatic birch BpubFinn407507_3I, C. symptomatic birch BpenGer407526_5M, D. symptomless birch BpubGer4 and E. symptomless birch BpenGerMO197542. Labels include taxon; number of reads assigned to taxon, summarized number of reads.

The taxonomic content of the obtained datasets, as provided by the BLAST analyses, was visualized using MEGAN [17]. The result of the BLAST analyses are parsed to assign the best hits to appropriate taxa in the NCBI taxonomy. As a result, the taxonomical content (“species profile”) of the sample from which the reads were collected was estimated, with a particular focus on viral species (Fig 2A–2E).

### Validation of the presence of novel viruses in birches

In order to confirm the presence of the identified novel viruses, specific RT-PCR assays were performed using virus-specific primers designed using the sequence of the scaffolds assembled for each agent (Table 1). These primer pairs were designed using OligoCalc [18] and, respectively, target regions within the RdRp domain (Carla for/rev; nt 926–1558) for the new carlavirus, within the methyl transferase (MTR) domain (RNA1) for the new idaeovirus (Idaeo_for/rev; nt 487–1060) and within the coat protein domain for the new capillovirus-sequence (Betaflexi_for/rev; nt 95–552).

**Table 1:** Primers used for genome completion and for the specific detection of the novel viruses.

Primer name	Primer sequence (5’– 3’)	Annealing temperature	Product length (bp)
**LD1_Carla_Ger526**	GGATGGTAATGGCAAATCGACCT	62°C	350
**LD prim**	CACTGGCGGCCGCTCGAGCATGTACT		
**5Race1-Carla-Ger407526**	GAAATCATGCTCTGCTCCGTGCTGGTG	72°C	188
**Carla_for**	CTTTGGTGCCGAATGAACGG	53 °C	632
**Carla_rev**	CACCGTCACCTTGGGCTATT		
**Idaeo_for**	GAGTTCGGGTGTTCGGTCTT	55 °C	573
**Idaeo_rev**	GGTGAACCGCCCAATCCTTA		
**Betaflexi_for**	CCGGCGATAAATCACGA	53 °C	457
**Betaflexi_rev**	AAAGGCCGTGGAAGACATGA		

Pooled samples of three to five leaves from different twigs of each seedling were used. The first-strand cDNAs were synthesized from 1 μg of total RNA in a 20 μl reaction volume of 1 x RT buffer (Thermo Scientific) containing 1 μM dNTPs mix, 200 U RevertAid Premium reverse transcriptase (Thermo Scientific), 20 U Ribolock RNase inhibitor (Thermo Scientific) and 100 pmol of random hexamer-oligonucleotides (Biomers.net GmbH). Subsequent PCR amplifications were conducted in a 25 μl volume of 1 x DreamTaq Buffer (Thermo Scientific) containing 0.2 μM dNTP mix, 0.625 U of DreamTaq DNA polymerase and 1 μM of each forward and reverse primer (Table 1). The thermal cycles were as follows: 2 min at 95 °C followed by 35 cycles at 95 °C for 30 s, T_anneal_ for 30 s, 72 °C for 40 s, with a final extension step of 72 °C for 5 min. Omitting the primers sequences, the amplified fragments are 592 nucleotides (nt) long for the carlavirus, 533 nt for the idaeovirus and 420 nt for the capillovirus. PCR products were directly submitted for Sanger sequencing (Macrogen) without previous cloning.

### Completion of the carlavirus genome ends

Assuming a dsRNA stage of the tentative carlavirus, 5’ and 3’ ends of the genome were determined using a 5’ Rapid amplification of cDNA-ends (5’ RACE) strategy, and a polyA-anchored Long Distance-RT-PCR, respectively. The 5’ RACE reaction was performed according to the kit manufacturer’s instructions (Clontech/Ozyme, Saint-Quentin en Yvelines, France) (Tprimer 5Race1-Carla-Ger407526; Table 1), and the 3’ genome end was amplified following the protocol described by Youssef et al. [19] (primers LD1_Carla_Ger526 and LD prim; Table 1).

### Phylogenetic analyses of carlavirus sequences

Multiple nucleotide or amino acid sequence alignments were performed as well as pairwise sequence identity calculations using AliView version 1.17.1 [20]. For the phylogenetic comparisons of complete RdRp and MP regions, all identified carlavirus species represented in GenBank to date were used. Bootstrapped Maximum Likelihood (ML) trees were constructed with MEGA6 [21]. Robustness of nodes of the phylogenetic tree was assessed from 1,000 bootstrap resamplings, and values >70% displayed for trees’ internal nodes.

## Results

### Birch metagenome taxonomic analysis with focus on the virome

The results obtained by MEGAN analysis regarding the taxonomic content of contigs assembled from the RNA-Seq reads are shown in Fig 2A–2E, together with the number of reads assigned to each taxon. For symptomatic sample BpenGer407526_5M, out of the 598.260 reads assessed, 561.584 belong to Eucaryota, most of them to the Phylum *Spermatophyta*—where *Betula sp*. is classified—and the rest belong to Protista (Alveolata) and Opisthokonta (Fungi and Vertebrata) (Fig 2C). From the 31.258 viral reads, 3.481 reads are attributed to birch leaf roll-associated virus (*Badnavirus*, *Caulimoviridae*) and specifically, to two variants of this virus (see ref. [11] for detailed description). Within the single-stranded RNA viruses, 24.516 reads belong to cherry leaf roll virus (*Nepovirus*, *Secoviridae*), while 2.835 reads are analyzed as representing agent(s) in the family *Betaflexiviridae*, with affinities to helleborus net necrosis virus (2.826 reads) and apple stem grooving virus (9 reads). The presence of 426 reads from Human mastadenovirus E (dsDNA viruses) is attributed to possible contamination of the sample during sample handlings or sequencing (all five samples exhibit presence of this human virus).

*B*. *pubescens* samples BpubFinn407501_3A and BpubFinn407507_3I are both found to be infected by the badnavirus BLRaV, respresented by a high number of reads (Fig 2A and 2B). Furthermore, in the sample BpubFinn407501_3A, 18 reads are attributed to hobart betaflexivirus 1, an unclassified member of the *Betaflexiviridae* family, and 51 reads to Totiviruses, known to infect fungi (Fig 2A). Both birch seedlings BpubGer4 and BpenGerM0197542, which did not show symptoms at the time of grafting and sampling, are negative for all viruses present in the symptomatic ones. However, 497 reads in the sample BpenGerMO197542 are attributed to the genus *Idaeovirus*, its closest relatives being blackcurrant leaf chlorosis-associated virus and privet leaf blotch-associated virus (Fig 2E).

An overview of the obtained RNA-seq data identified in each sample is presented in Table 2.

**Table 2:** Virome data generated for each birch seedling. The number of reads and their percentage in the sample, as well as the validation output through RT-PCR (+/-), are presented. (BLRaV: birch leafroll-associated virus; CLRV: cherry leaf roll virus; BiCV: birch carlavirus; BCV: birch capillovirus; BIV: birch idaeovirus).

	Bpub Finn 407501_3A			Bpub Finn 407507_3I			BpenGer 407526_5M			Bpen Ger MO197542			Bpub Ger4		
	Number of reads	%	PCR	Number of reads	%	PCR	Number of reads	%	PCR	Number of reads	%	PCR	Number of reads	%	PCR
**Total**	803.120			613.923			725.231			546.722			682.408		
**BLRaV**	3211	0.4	+	5567	0.9	+	3529	0.49	+	1	0.0002	-	1	0.00015	-
**CLRV**	3	0.0004	+	3	0.0005	+	10896	1.5	+	0	0	-	2	0.0003	-
**BiCV**	3	0.0004	-	5	0.0008	-	2881	0.397	+	2	0.00037	-	1	0.00015	-
**BCV**	21	0.0026	+	3	0.0005	+	20	0.003	+	5	0.0009	+	2	0.00029	+
**BIV**	0	0	-	0	0	-	0	0	-	195	0.036	+	0	0	-

### Full genome assembly of a new birch CLRV variant

CLRV was detected by HTS in only one of the tested symptomatic birches, the *B*. *pendula* BpenGer407526_5M from Berlin. The full-length genome, which consists of two RNA segments, was assembled. RNA1 is 7,848 bp-long and highly similar to the birch isolate already deposited in GenBank (LT883167, 96% nt identity). RNA2 is 6,459 bp-long and exhibits a lower level of identity with the birch CLRV isolate (LT883166, 91% nt identity), and is similar to the identity level observed with the cherry CLRV isolate (JN104385, 91% nt identity). The genomic sequences of this new CLRV variant have been deposited in GenBank under accession numbers MK402281 (Cherry leaf roll virus, isolate CLRV_BpenGer407526 segment RNA1, complete genome) and MK402282 (Cherry leaf roll virus, isolate CLRV_BpenGer407526 segment RNA2, complete genome).

### Partial genome assembly of a novel idaeovirus

In the dataset from the seedling BpenGerMO197542, two long contigs of an uncharacterized virus with affinities to idaeoviruses were assembled (see Fig 2E). The first contig is 5,232 bp-long and encodes a putative protein, which in the BLASTP analysis shows high level of identity with the ORF1 of the blackcurrant leaf chlorosis-associated virus (BCLCaV, YP_009361854, 63% aa identity)—a novel, recently described idaeovirus [22]. The ORF1 initiates at nt position 283–285 of the contig and codes for a 1649 aa putative replication-associated protein with conserved methyltransferase (MTR), helicase (HEL) and RNA-dependent RNA polymerase (RdRp) domain. However, the protein is not complete as a stop codon is not reached and amino acids of the C-terminal end of the protein are missing compared to BCLCaV. The second contig is 1,595 bp-long and harbours 2 ORFs. The first ORF initiates at nt position 5–7 of the contig, ends at positions 1091–1093, and encodes a putative 362 aa-long movement protein exhibiting identities with the corresponding protein of BCLCaV (YP_009361835, 43% aa identity). The second ORF of the RNA2 initiates at nt positions 1090–1092, overlapping with the stop codon of the first ORF, as is also observed for BCLCaV [23]. This second ORF encodes a putative coat protein (CP), which is homologous with the CP of BCLCaV (YP_009361836, 47% aa identity). However, only the first 167 aa of the protein are available, as the genome is not completely covered by the contig. The presence of this novel virus was validated by RT-PCR with specific primers (Idaeo_for/Idaeo_rev; Table 1) in the tested seedling. Sequencing of the amplified products provided a sequence identical to that of the original contig, thus further confirming the infection. We suggest, therefore, that the two contigs correspond to a novel idaeovirus, tentatively named birch idaeovirus (BIV). The obtained incomplete viral sequences were deposited into the GenBank databases under accession numbers MK402235 (Birch idaeovirus, isolate BpenGerMO197542 segment RNA1, partial cds) and MK402236 (Birch idaeovirus, isolate BpenGerMO197542 segment RNA2, partial cds). Interestingly, we recently identified 3 contigs with ca. 98% identity with the novel idaeovirus sequence reported here in a large *B*. *pendula* pollen RNA-Seq dataset (SRX2768860).

**Fig 3:**
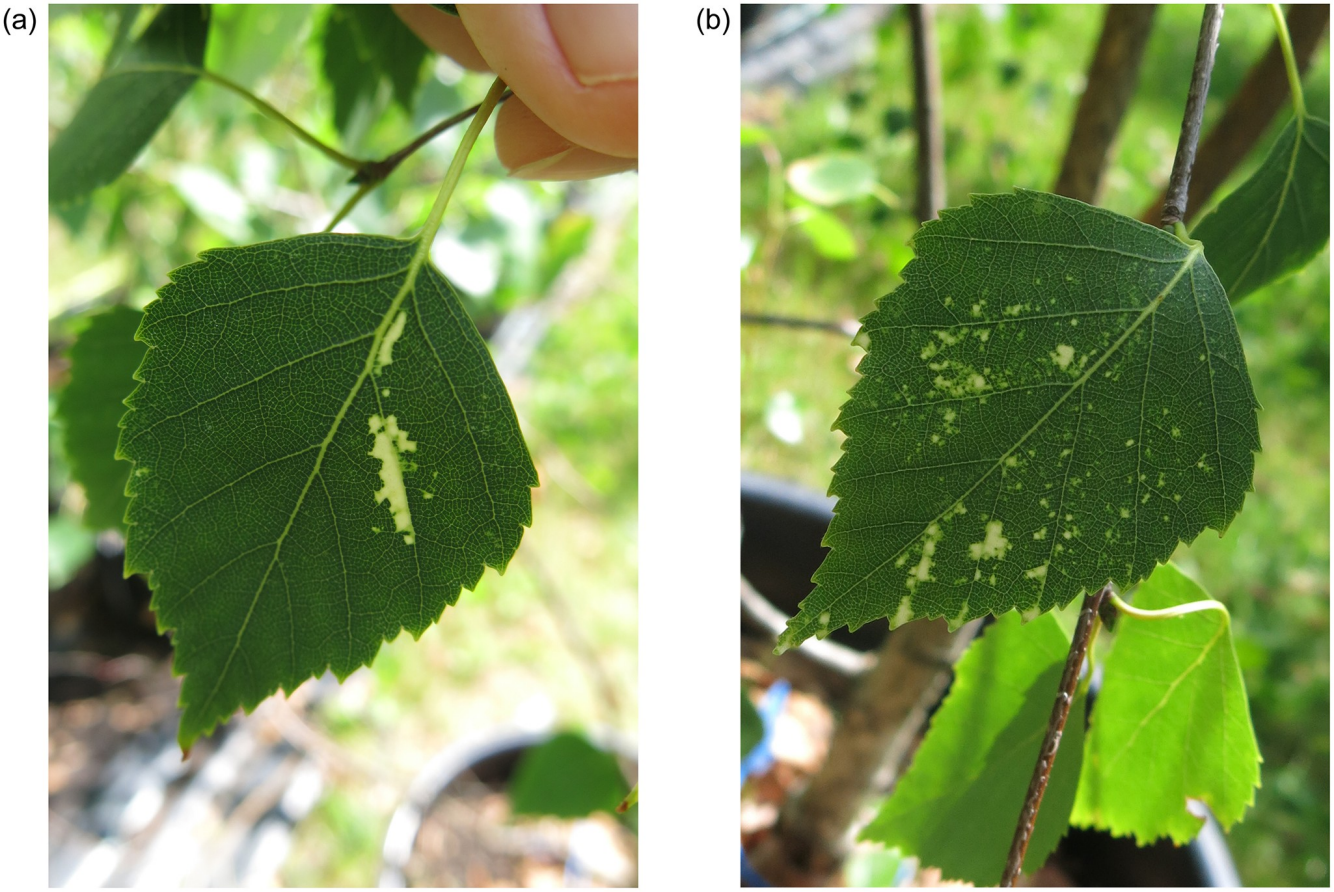
Symptoms appeared in the seedling BpenGerMO197542.

In August 2015, virus-like symptoms were detected on seedling BpenGerMO197542 (Fig 3). White-yellowish blotches appeared sporadically on a few leaves of the seedling. Further investigations shall clarify, if these symptoms are caused by the new ideaovirus.

### Assembly of a capillo-like virus sequence

From the RNA-seq dataset of the BpenGer407526_5M seedling, an 821 bp-long contig was assembled, which exhibits identities with apple stem grooving virus (ASGV, *Betaflexiviridae*, *Capillovirus*, Fig 2C). Further attempts to obtain longer sequences of the novel virus by means of PCR resulted in a 1114 bp-long contig extending all the way to the 3’-poly(A) tail. This contig was submitted to GenBank under accession number MK402233 (Birch capillovirus, isolate BpenGer407526_5M, partial sequence). Within the contig, the 250 aa-long (753 nt) coat protein sequence of this novel virus (nt positions 60–812) is encoded. In the BLASTP analysis, this putative protein shares low but significant identity with the CP of ASGV (AFH75121, 30% identity). Furthermore, a 597 bp-long sequence covering part of the same genomic region was assembled from the BpubFinn407501_3A reads (accession number MK402234, Birch capillovirus, isolate BpubFinn407501_3A, partial sequence), showing 98,7% nt identity with the first one. This contig is assembled from the reads attributed to hobart betaflexivirus 1 in the Megan analysis of Fig 2A.

**Fig 4:**
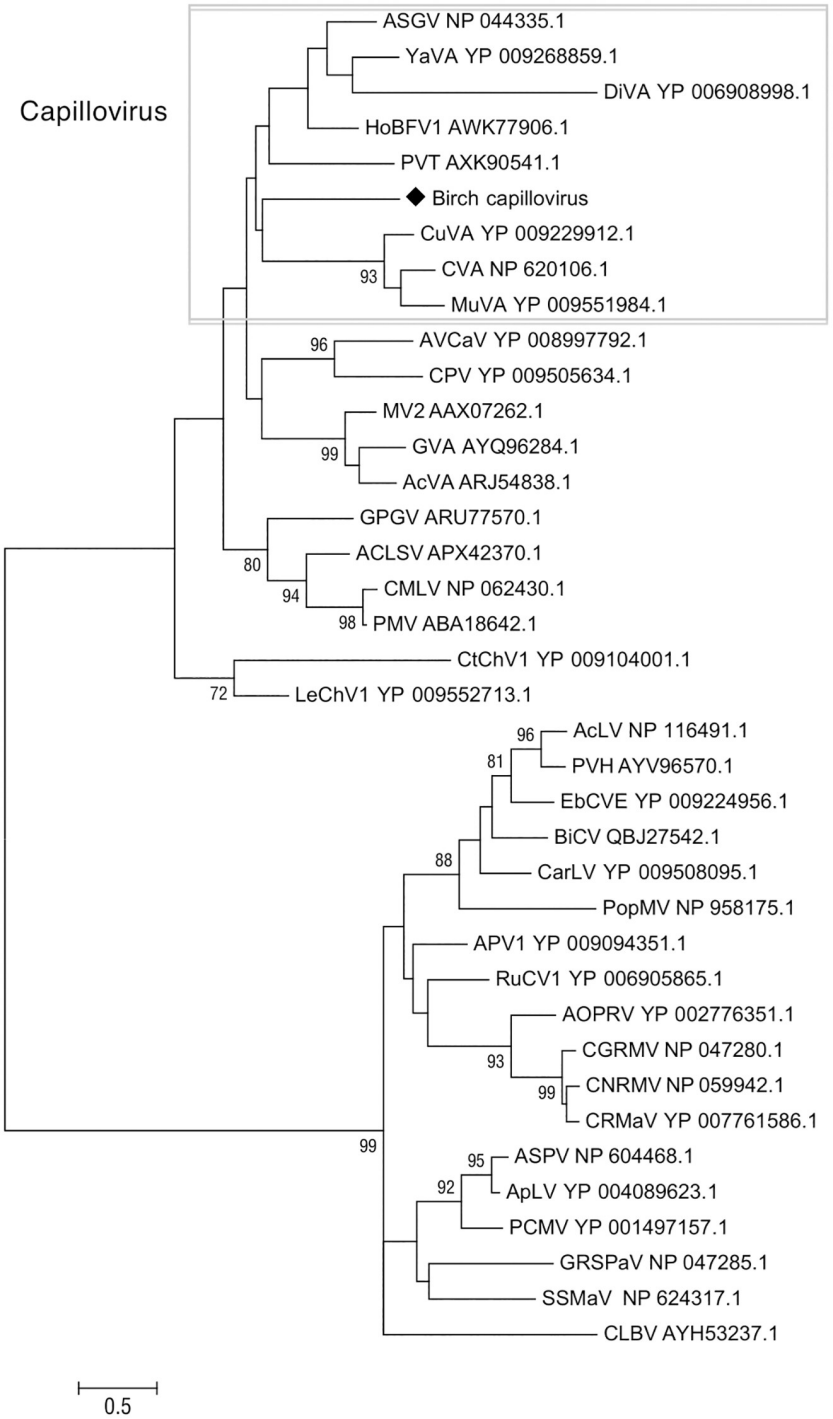
Phylogenetic tree reconstructed using the amino acid sequences of the CP of *Betaflexiviridae* members. The tree was reconstructed using the Maximum Likelihood method and the statistical significance of branches was evaluated by bootstrap analysis (1,000 replicates). Only bootstrap values above 70% are indicated. The scale bar represents 5% amino acid divergence. Members of the *Capillovirus* genus are indicated within the rectangle. Virus abbreviations and accession numbers are as follows: apple stem grooving virus (ASGV), yacon virus A (YaVA), diuris virus A (DiVA), hobart betaflexivirus 1 (HoBFV1), currant virus A (CuVA), cherry virus A (CVA), mume virus A (MuVA), potato virus T (PVT), apricot vein clearing associated virus (AVCaV), caucasus prunus virus (CPV), mint virus 2 (MV2), grapevine virus A (GVA), actinidia virus A (AcVA), grapevine Pinot gris virus (GPGV), apple chlorotic leaf spot virus (ACLSV), cherry mottle leaf virus (CMLV), peach mosaic virus (PMV), carrot Ch virus 1 (CtChV1), lettuce Chordovirus 1 (LeChV1), aconitum latent virus (AcLV), potato virus H (PVH), elderberry carlavirus E (EbCVE), birch carlavirus (BiCV), carnation latent virus (CarLV), poplar mosaic virus (PopMV), asian prunus virus 1 (APV1), rubus canadensis virus 1 (RuCV1), african oil palm ringspot virus (AOPRV), cherry green ring mottle virus (CGRMV), cherry necrotic rusty mottle virus (CNRMV), cherry rusty mottle-associated virus (CRMaV), apple stem pitting virus (ASPV), apricot latent virus (ApLV), peach chlorotic mottle virus (PCMV), grapevine stem pitting-associated virus (GRSPaV), sugarcane striate mosaic-associated virus (SSMaV) and citrus leaf blotch virus (CLBV).

As the encoded proteins of the new viral sequence show less than 80% aa identity with CP sequences from other capilloviruses, they are suggested to belong to a novel species of the genus *Capillovirus*. To investigate the assumption that the new virus is closely related to other capilloviruses, the phylogenetic relationships of the CP protein sequences from members of the *Betaflexiviridae* family were analyzed. In the obtained ML and NJ trees, the new viral sequence reliably clustered within the capilloviruses clade (Fig 4). In conclusion, the low amino acid identity with members of *Capillovirus*, as well as the phylogeny generated for the CP regions, suggest that this viral sequence belongs to the genus Capillovirus and is, therefore, tentatively named birch capillovirus (BCV).

The presence of the capillovirus sequence was confirmed by RT-PCR not only in the seedling Bpen5MGer407526_5M from which it originated, but also, in all four other seedlings analyzed here (symptomatic and not symptomatic) and in other trees from Berlin and Rovaniemi [24].

### Full genome assembly of a novel carlavirus from birch

BLASTN and BLASTP annotation of the assembled contigs from symptomatic birch BpenGer407526_5M revealed one large contig exhibiting high BLAST scores with members of the genus *Carlavirus* (*Betaflexiviridae*) (see Fig 2C, reads attributed by MEGAN to helleborus net necrosis virus). This 8,846 nt contig covers a near-complete carlaviral genome, missing only the ends.

PolyA-anchored long-Distance (LD)-PCR and 5’ RACE enabled the completion of the genome by generating sequences that perfectly matched the contig in the overlap regions. The presence of the virus was confirmed by specific RT-PCR performed in the seedling in which it was first detected in tree BpenGer407526_5M and in other trees in Berlin [24]. The full-length genomic sequence of this novel agent has been deposited in GenBank under accession number MH536506 (Birch carlavirus, isolate BpenGer407526_5M, complete genome).

**Fig 5:**
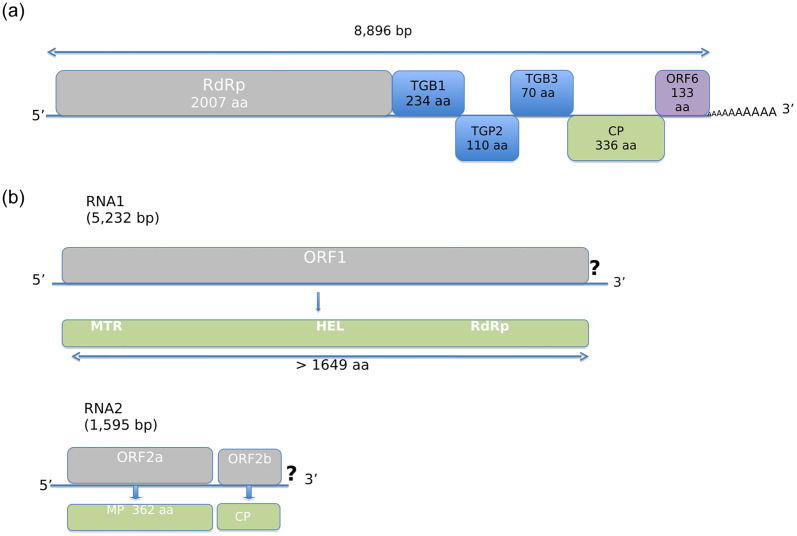
Schematic representation of the genome organization of the novel *Birch carlavirus* (BiCV; Fig 5A) and *Birch idaeovirus* (BIV; Fig 5B).

The genome of this virus is 8,896 base pairs (bp) long, which is similar to the genome size of typical carlaviruses (8,3–8,7 kb) [25]. It shows a typical Carlavirus organization with 6 ORFs, including an RNA-dependent RNA polymerase (RdRp; nt 61–6,084), three triple gene block proteins (TGB1; nt 6,153–6,857, TGB2; nt 6,835–7,167, TGB3; nt 7,169–7,381), a coat protein (CP; nt 7,432–8,442) and a nucleic acid binding protein (NABP, ORF6; nt 8,442–8,843) (Fig 5). In the BLASTP analysis, the RdRp shows identities with the corresponding protein of Carlaviruses, the closest being helleborus net necrosis virus (47% identity). The CP protein also shows significant levels of aa identity (41%–55%) with other carlaviral CPs (see also phylogenetic analysis below), the closest being elderberry carlavirus B (55% identity). The TGB proteins also have the closest affinities to various carlaviruses: 53% identity for the TGB1 (elderberry carlavirus A), 54% for the TGB2 (poplar mosaic virus) and 71% for the TGB3 (carrot carlavirus). The NABP encoded by ORF6 is most similar to the corresponding protein of helleborus mosaic virus (42% identity).

### Phylogenetic analysis of the novel carlavirus

**Fig 6:**
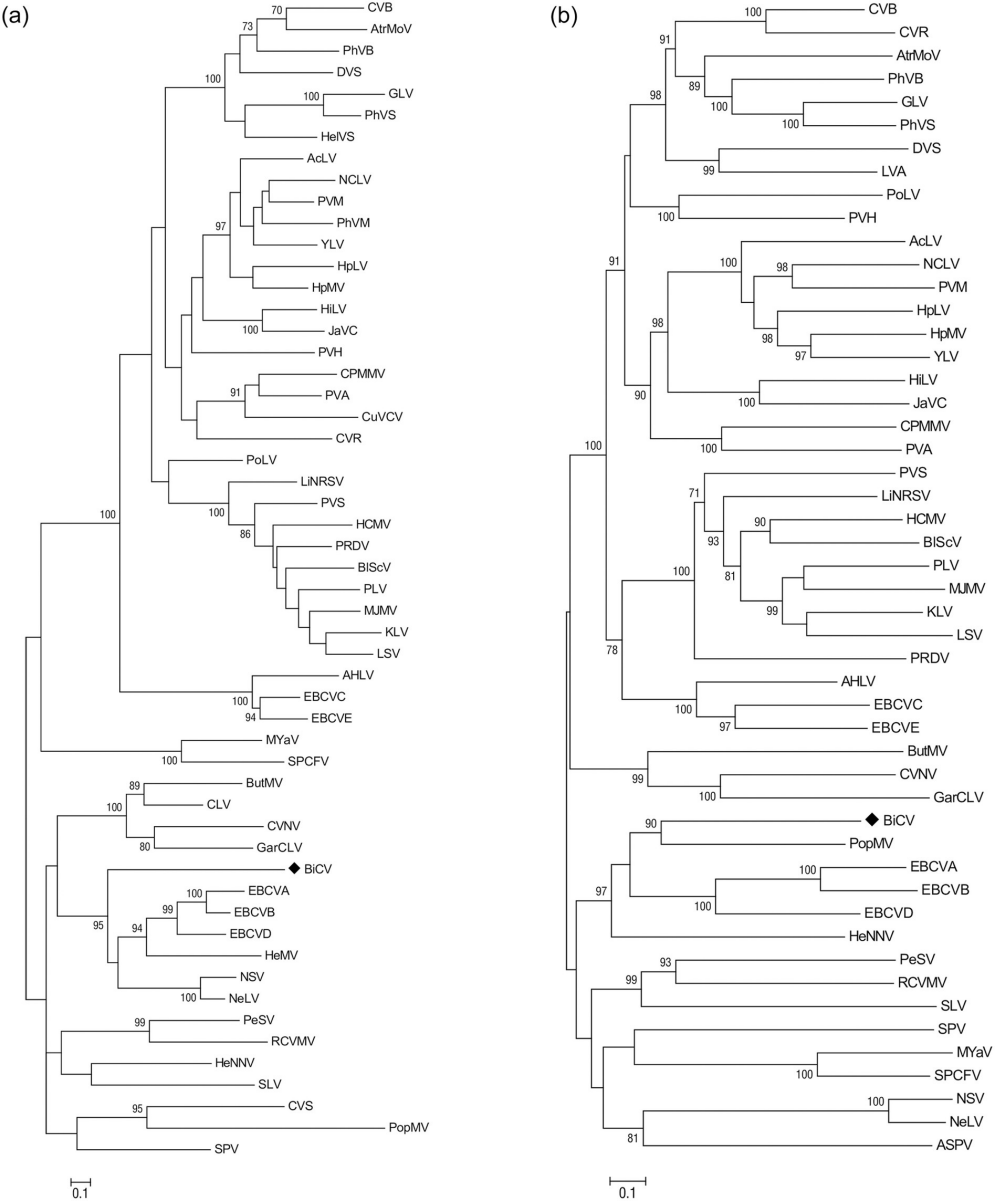
Phylogenetic trees reconstructed using the amino acid sequences of the CP (A) and the RdRp (B) of carlaviruses. The tree was reconstructed using the Maximum Likelihood method and the statistical significance of branches was evaluated by bootstrap analysis (1,000 replicates). Only bootstrap values above 70% are indicated. The scale bar represents 10% amino acid divergence. Virus abbreviations and accession numbers are as follows: aconitum latent virus (AcLV, NC_002795.1), american hop latent virus (AHLV, NC_017859.1), apple stem pitting virus (ASPV, NC_003462.2), atractylodes mottle virus (AtrMoV, KR349343.1), birch carlavirus (BiCV), blueberry scorch virus (BlScV, NC_003499.1), butterbur mosaic virus (ButMV, NC_013527.1), carnation latent virus (CLV, AJ010697.1), carrot virus S (CVS, EU881919), chrysanthemum virus B (CVB, NC_009087.2), chrysanthemum virus R (CVR, MG432107.1), coleus vein necrosis virus (CVNV, NC_009764.1), cowpea mild mottle virus (CPMMV, NC_014730.1), cucumber vein-clearing virus (CuVCV, JN591720.1), daphne virus S (DVS, NC_008020.1), elderberry carlavirus A (EBCVA, NC_029085.1), elderberry carlavirus B (EBCVB, NC_029086.1), elderberry carlavirus C (EBCVC, NC_029087.1), elderberry carlavirus D (EBCVD, NC_029088.1), elderberry carlavirus E (EBCVE, NC_029089.1), gaillardia latent virus (GLV, NC_023892.1), garlic common latent virus (GarCLV, NC_016440.1), helenium virus S (HelVS, D10454.1), helleborus mosaic virus (HeMV, FJ196838.1), helleborus net necrosis virus (HeNNV, NC_012038.1) hippeastrum latent virus (HiLV, NC_011540.1), hop latent virus (HpLV, NC_002552.1); hop mosaic virus (HpMV, NC_010538.1), hydrangea chlorotic mottle virus (HCMV, NC_012869.1), jasmine virus C (JaVC, NC_030926.1), kalanchoe latent virus (KLV, NC_013006.1), ligustrum necrotic ringspot virus (LiNRSV, NC_010305.1), ligustrum virus A (LVA, NC_031089.1), lily symptomless virus (LSV, NC_005138.1), melon yellowing-associated virus (MYaV, LC224308.1), mirabilis jalapa mottle virus (MJMV, NC_016080.1) narcissus common latent virus (NCLV, NC_008266.1), narcissus symptomless virus (NSV, NC_008552.1), nerine latent virus (NeLV, NC_028111.1), passiflora latent virus (PLV, NC_008292.1), pea streak virus (PeSV, NC_027527.1), pepper virus A (PVA, NC_034376.1), phlox virus B (PhVB, NC_009991.1), phlox virus M (PhVM, FJ159381.1), phlox virus S (PhVS, NC_009383.1), poplar mosaic virus (PopMV, NC_005343.1), potato latent virus (PoLV, NC_011525.1), potato virus H (PVH, NC_018175.1), potato virus M (PVM, NC_001361.2), potato virus S (PVS, NC_007289.1), potato rough dwarf virus (PRDV, NC_009759.1), red clover vein mosaic virus (RCVMV, NC_012210.1), shallot latent virus (SLV, NC_003557.1), sweet potato chlorotic fleck virus (SPCFV, NC_006550.1), sweet potato virus (SPV, NC_018448.1) and yam latent virus (YLV, NC_026248.1).

Phylogenetic relationships between the birch carlavirus and the sequences of carlaviruses known to date were estimated based on amino acid sequences comparisons. The topology of the trees was similar, irrespective of whether the ML or NJ algorithms were used. Fig 6 shows representative ML trees obtained using the CP or RdRp protein sequences. Using the CP sequence, the birch virus clusters with woody host carlaviruses: elderberry carlaviruses A, B and D (EBCVA, EBCVB, EBCD) and, more distantly, PopMV are shown. The RdRp clusters together with poplar mosaic virus (PopMV) and elderberry carlavirus A, B and D (EBCVA, EBCVB, EBCD). The new virus is clearly only distantly related phylogenetically to all carlaviruses currently represented in the GenBank sequence database (Fig 6), and exhibits less than 80% aa identity with the CP or RdRps of known carlaviruses. Similar results are obtained from the proteins encoded by the triple gene block (TGB1, TGB2, TGB3) and the ORF6 sequence (S1a–S1d Fig). Taken together, these results demonstrate that it represents a new member of the genus *Carlavirus* and it is tentatively named birch carlavirus (BiCV).

## Discussion

Taken together, the HTS analyses revealed distinguishable virome of the individual birch trees. In the case of the BLRD BpenGer407526_5M sample, the virome comprises five viral agents, namely a new isolate from the well-characterized CLRV nepovirus, two variants of the recently discovered birch leafroll-associated badnavirus (BLRaV), an isolate of the newly discovered birch carlavirus (BiCV), as well as a capillovirus sequence (BCV) (Fig 2C). In the case of the BLRD *B*. *pubescens* seedlings, the virome is less complex; in BpubFinn407501_3A, the presence of a BLRaV and a BCV variant was shown (Fig 2A), while in BpubFinn407507_3I, a single BLRaV-variant was found (Fig 2B). The virome of the control seedlings was simple. While in the BpenGerMO197542 only the novel idaeovirus BIV could be identified, in the seedling BpubGer4 no virus could be detected.

Based on these findings, new scenarios for the disease etiology can be proposed. The initial hypothesis implicating *Cherry leaf roll virus* (CLRV) as the only BLRD causal agent [12, 15] is not supported by the new data. BLRD symptoms were observed also in trees free of CLRV infection. Due to BLRaV detection in the virome of symptomatic seedlings and its absence from symptomless ones, we suggest that this virus could alone cause the disease (in the case of BpubFinn407507_3I, it is the only virus detected with a significant number of reads). The mix-infections observed in individual BLRD plants make it hard to determine the etiology unambiguously. The other novel virus, BiCV, was detected in only one of the three symptomatic seedlings (in mixed infection with CLRV and BLRaV). We suggest that it cannot be solely responsible for BLRD but could possibly still contribute in cases of mixed infection with BLRaV and/or CLRV. This is however only a hypothesis. Alternatively, BiCV might not be significant for the observed symptomatology. Concerning the ideaovirus (BIV), it was only detected in a seedling without leaf-roll and chlorotic vein banding symptoms -typically associated with the BLRD-, ruling out its involvement in the disease. The BCV capillovirus sequence was detected in symptomatic and non-symptomatic seedlings and it is tempting to hypothesize that it does not contribute to symptoms. Overall the current results are compatible with a scenario making BLRaV the prime etiological agent for BLRD, without excluding a potential additional contribution from CLRV or BiCV.

The use of HTS has revealed that mixed infections are frequent in birch, involving different viruses. The complex virome observed in the tested birch seedlings has been repeatedly observed in more birches in Berlin in other studies. In an investigation of viruses’ distribution in urban parks in Berlin for two consecutive years, BiCV was present in 16% of the tested birches [26]. In five of these seedlings, a co-infection of BiCV, CLRV and the BLRaV was found. Co-infection with BiCV, CLRV, ApMV (*Apple mosaic virus*) and BLRaV in birches was observed in urban landscapes in Berlin, with an incidence in symptomatic leaves of around 29% within three years of investigation. These data indicate that mixed infections in birch are widespread. It is not easy to establish a correlation between such viral complexes and the appearance of symptoms or to differentiate symptomatology in cases of infection by a single virus or by two virus species [24]. In contrast to annual plants, in a large volume of birch trees, the symptomatology may differ in different parts of the canopy and is presumably related to differences in the virus population—as recently demonstrated in birches [13]—or to other parameters.

In light of a holistic understanding of the disease pathogenesis, the “pathobiome” concept has been developed, which represents the pathogenic agents integrated within its biotic environment [27]. Understanding the pathobiome thus requires (1) an accurate knowledge of the microorganism community, (2) clear evidence of any effect(s) this microorganism community has on pathogenesis, (3) an understanding of the impact of the microorganism community on persistence, transmission and evolution of pathogenic agents, and (4) knowledge of biotic and abiotic factors that may disrupt the pathobiome and lead to the onset of pathogenesis. According to this concept, the diverse nature of the viruses detected in birches may play an important, but still to be clarified role in disease development, as each virus may interact with or disturb the virome, ultimately causing disease [27].

Apart from the virome that is described in detail here, attention should also be given to the remaining microorganisms detected in the samples. Bacterial species (*Proteobacteria*, *Terrabacteria*, FCB Group bacteria), unclassified Totiviruses, as well as thousands of unassigned reads, are part of the birch metagenome. Our findings should be examined under the holistic view of the “hologenome theory” [28], which proposes that plants must not be viewed as autonomous entities but rather as holobionts, within which all interacting organisms contribute to the overall stability of the system [29]. Driving factors such as microbiota in the soil, the rhizosphere, the rhizoplane, the endosphere and the aboveground compartment play significant roles in the health status of the holobiont [30]. With our study, we provide some new data regarding the birch microbiota complexity. Their role is not analysed in the present study, but they can be combined with further data in the future.

Concerning the new capillovirus sequence, given the short length of the genomic region characterized, there are still some doubts regarding whether the detected sequences indeed represent an existing virus and, if so, whether this virus can unambiguously be assigned to the *Capillovirus* genus. This can only be sorted out, ultimately, by efforts to obtain the full genome of the suspected virus. It is noteworthy, that a 600-nt sequence with very high identity with the BCV contig MK402234 has been identified within the transcriptomic data generated from pollen of *Betula verrucosa* [31]. This indicates that if indeed the sequence identified here is viral, the agent might be more broadly present in other *Betula* species.

To our knowledge, it is the first time that metagenome data of a forest tree species (*Betula sp*.) are reported. In comparison to cultivated plants, few measures have been taken regarding knowledge on viruses present in forest ecosystems. Missing data or unawareness concerning viral incidence in forests may lead to unjustified disease diagnosis and determination of the causal agent. It is not only birch that suffers from viral diseases, as virus-like symptoms are commonly observed in *Fraxinus* sp. (Central Europe, Switzerland, Germany), in *Quercus* sp. (Germany, Sweden, Romania), in *Ulmus* sp. (Germany, Sweden, Gotland), in *Acer* sp. (Germany), in *Populus* sp. (Germany, Finland), and in *Sorbus* sp. (Germany, North and Central Europe) [16]. Based on extended experience on recognising symptomatology of viral causal agents and on monitoring the distribution of viral diseases, it is suggested that viral infections alter plant predisposition and affect the health status of many forest and urban trees. HTS technologies may offer a deeper investigation of the viruses in forest species and supply more knowledge concerning the virome of a forest. Past [32] and future studies are expected to enlighten the possible modes of virus interaction with abiotic and biotic factors in forest and urban trees.

## Supporting information

S1 Fig Phylogenetic trees reconstructed using the amino acid sequences of the proteins TGB1 (A), TGB2 (B) and TGB3 (C) encoded by the triple gene block and the protein encoded by ORF6 (D) of carlaviruses. The trees were reconstructed using the Maximum Likelihood method and the statistical significance of branches was evaluated by bootstrap analysis (1,000 replicates). Only bootstrap values above 70% are indicated. The scale bar represents 10% amino acid divergence.
(TIF)
