# Supplementary figures and images for: Unravelling the virome in birch: RNA-Seq reveals a complex of known and novel viruses
Source: PLoS One. 2020 Jun 26;15(6):e0221834. doi: 10.1371/journal.pone.0221834 (PMC7319284; doi:10.1371/journal.pone.0221834)

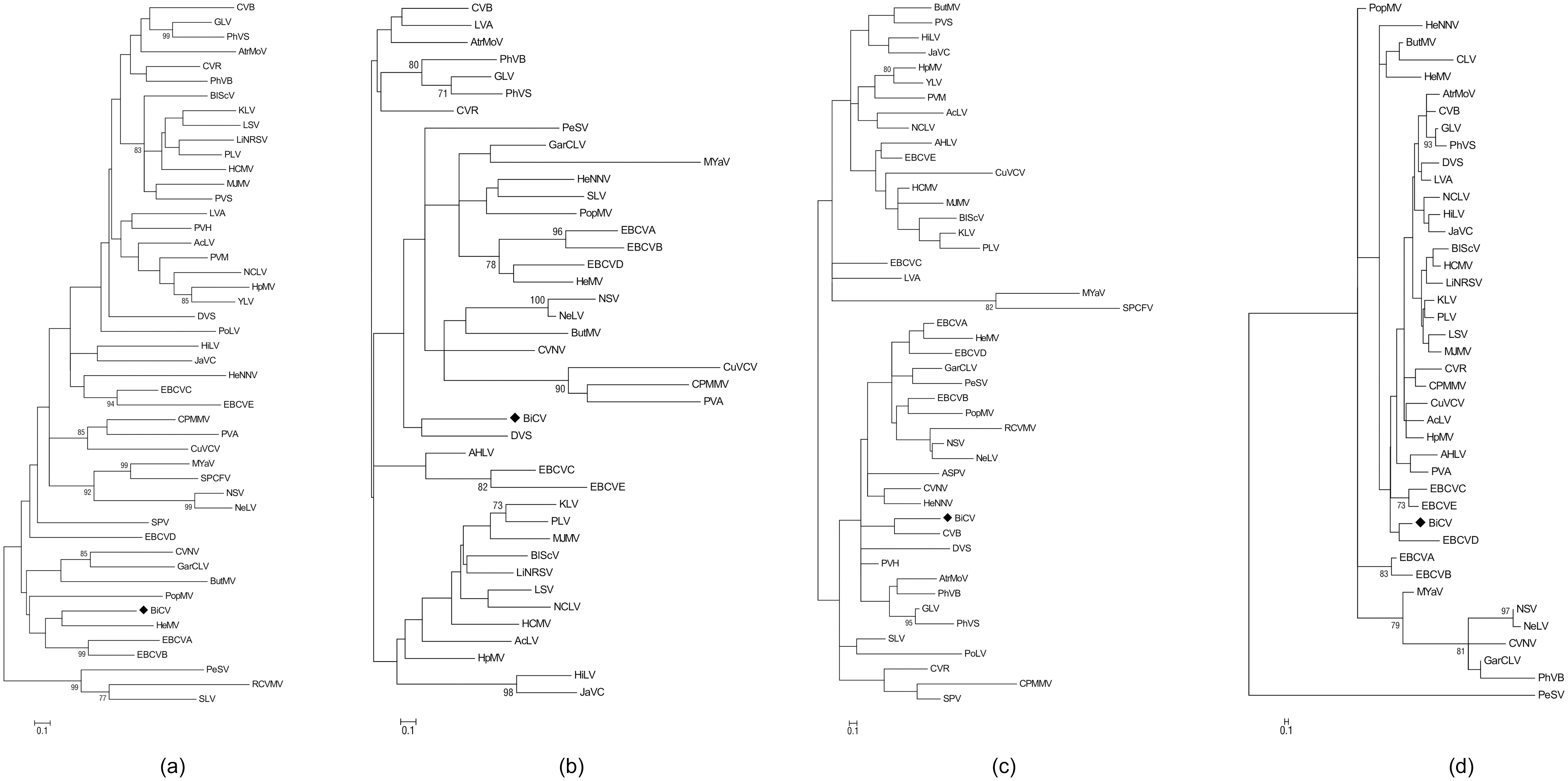

Supplement: S1 Fig — The trees were reconstructed using the Maximum Likelihood method and the statistical significance of branches was evaluated by bootstrap analysis (1,000 replicates). Only bootstrap values above 70% are indicated. The scale bar represents 10% amino acid divergence. (TIF) [file pone.0221834.s001.tif]
